# The expression of the surfactant proteins SP-A and SP-B during postnatal alveolarization of the rat lung

**DOI:** 10.1371/journal.pone.0297889

**Published:** 2024-03-14

**Authors:** Franziska Roeder, Lars Knudsen, Andreas Schmiedl

**Affiliations:** 1 Institute of Functional and Applied Anatomy, Medical Hannover School, Hannover, Germany; 2 Biomedical Research in Endstage and Obstructive Lung Disease Hannover (BREATH), Member of the German Center for Lung Research (DZL), Hannover, Germany; Kurume University School of Medicine: Kurume Daigaku Igakubu Daigakuin Igaku Kenkyuka, JAPAN

## Abstract

**Objective:**

Surfactant-specific proteins (SP) are responsible for the functional and structural integrity as well as for the stabilization of the intra-alveolar surfactant. Morphological lung maturation starts in rat lungs after birth. The aim of this study was to investigate whether the expression of the hydrophilic SP-A and the hydrophobic SP-B is associated with characteristic postnatal changes characterizing morphological lung maturation.

**Methods:**

Stereological methods were performed on the light microscope. Using immunohistochemical and molecular biological methods (Western Blot, RT-qPCR), the SP-A and SP-B of adult rat lungs and of those with different postnatal developmental stages (3, 7, 14 and 21 days after birth) were characterized.

**Results:**

As signs of alveolarization the total septal surface and volume increased and the septal thickness decreased. The significantly highest relative surface fraction of SP-A labeled alveolar epithelial cells type II (AEII) was found together with the highest relative SP-A gene expression before the alveolarization (3th postnatal day). With the downregulation of SP-A gene expression during and after alveolarization (between postnatal days 7 and 14), the surface fraction of the SP-A labeled AEII also decreased, so they are lowest in adult animals. The surface fraction of SP-B labeled AEII and the SP-B gene expression showed the significantly highest levels in adults, the protein expression increased also significantly at the end of morphological lung maturation. There were no alterations in the SP-B expression before and during alveolarization until postnatal day 14. The protein expression as well as the gene expression of SP-A and SP-B correlated very well with the total surface of alveolar septa independent of the postnatal age.

**Conclusion:**

The expression of SP-A and SP-B is differentially associated with morphological lung maturation and correlates with increased septation of alveoli as indirect clue for alveolarization.

## Introduction

The precondition for perfect breathing after birth is a morphologically mature lung with a sufficiently large gas exchange surface and an intact surfactant system for reducing the surface tension at the gas-liquid boundary of the distal airways [1,2]. The development of the lung can be divided into a prenatal and postnatal section. The prenatal section comprises the embryonic phase and the fetal phase. The fetal phase starts with the pseudoglandular stage, in which the epithelial sprouts continue to branch out and make the lungs appear like a tubulo-alveolar gland [3,4]. Further branching and distal widening of tubuli, epithelial differentiation of the distal ends of the widened distal ducts into alveolar epithelial cells type I and type II and increased capillarization displace more and more the mesenchyme in the canalicular stage [3,4]. The saccular stage is characterized by formation of sacculi with thick primary septa lined by alveolar epithelium. The primary septa are characterized by a lot of connective tissue and a double layered capillary bed [3,4]. In the alveolar stage, secondary septa are formed leading to an increase of the gas exchange region. The septa get thinner and a single layer capillary bed is formed. The duration of each developmental phase and the state of differentiation of the lungs at birth depends on the species and the postnatal behavior of the young. The sequence and morphology of the lung development stages is congruent in all mammals [3]. Therefore, most knowledge about lung development results from animal models, predominantly in mice. Because alveolarization in altricial species takes place after birth, rodents are also suitable for investigation of human diseases with delayed lung development such as bronchopulmonary dysplasia [5–8].

The alveolarization in human starts prenatally and continues after birth until adulthood [9]. Unlike humans, rats are born with morphologically immature lungs [9,10], that are still in the saccular phase [11]. Bulk alveolarization starts at postnatal day 4 and continues until the end of the third week. During alveolarization airspaces get smaller and septation of the distal airspaces increase by forming new secondary septa [9]. At the end of the third week the vascular maturation leads to a single layered capillary bed [12]. Bulk alveolarization within the first 3 weeks results in a very strong increase of the alveolar surface [13–16]. However, some alveolarization continues until postnatal day 60 (continued alveolarization) [10]. Although rat pups contain a morphologically immature lung, the surfactant system has to be mature enough to facilitate breathing and to prevent alveolar collapse by reducing the surface tension of the terminal airspaces [17,18]. Surfactant proteins and lipids are synthesized, secreted and recycled by alveolar epithelial cells type II (AEII) [19,20]. The differentiation of AEII and the occurrence of surfactant in lungs were verified already during prenatal development [19–24]. It is already known that in the late gestation period increased synthesis of surfactant lipids and proteins occurred [19]. So mRNAs for SP-A, B and C are already prenatally present in the distal portions of the ductal system in lung tissue of rats at 13 days gestation during the pseudoglandular period [24]. The synthesis of surfactant starts in the canalicular phase and therefore at a time when 80% of the wearing time has already passed [25]. Although rats are born in the saccular phase of lung development, which correspond to preterm human in the 23th gestation week, the surfactant has to be mature enough to prevent collapse of airspaces during deflation and expansion of distal airways during inflation. The surfactant proteins play an important role for formation, spreading and stabilization of the surfactant film as well as for inflammation silencing [26]. Particularly SP-A is involved in modifying surfactant homeostasis and is a part of the innate host response to environmental challenges and protect the lung from oxidative stress [27,28]. SP-B is crucial for the formation and the maintenance of the continuous spread of the surfactant film [29].

Up to now, there is no systematic evaluation of the relationship between morphological (alveolarization) and functional lung maturation including the surfactant system. Therefore, the aim of this study was to verify alveolarization using stereological methods and to examine whether the postnatal morphological maturation of the rat lung is associated with characteristic changes in surfactant protein synthesis, especially of SP-A and SP-B.

## Material and methods

F344 rats of either sex were kept under specific pathogen- and germ-free conditions. Furthermore, they had ad libitum access to food and water. Experiments had been approved by the Nds. State Office for Consumer Protection and Food Safety (2015/87) and met the NIH Guidelines for the Care and Use of Laboratory Animals [NIH Publication No. 85. reprint 2002].

### Processing of lungs

We investigated lungs of rats with different postnatal ages (3, 7, 14 and 21 days old) and adult rats (90 days).

The animals were anesthetized with isoflurane under a glass bell. Under deep anesthesia, the animal’s abdominal cavity was immediately opened and the animal was drained of blood through a huge incision in the abdominal aorta, leading to the animal´s death. Following thoracotomy, the heart and the lungs were visible and the right ventricle was cannulated. The lungs were then flushed with NaCl solution via the pulmonary artery. The left atrial appendage was cut to relieve the volume pressure [30]. After flushing, the heart was clamped to prevent blood from flowing back into the lungs. For histological studies, a cannula was inserted via the larynx, advanced into the trachea and tied. The heart lung block was carefully removed for further processing.

### Molecular biology

For protein and RNA isolation, the rinsed lungs were cut into pieces using a razor blade. From each area of the lungs approximately 3 mm blocks were taken, shock-frozen in liquid nitrogen and stored at -80°C until further protein and RNA isolation.

#### Western blotting of surfactant proteins

Approximately 20 mg of lung tissue per sample together with 300 ml of RIPA Lysis buffer (EMD Millipore) and one tablet of protease inhibitor (cOmplete Tablets Mini EASYpack, Roche, Switzerland) were lysed in the cell lysator for 2 minutes to isolate proteins. After cooling in an ice bath and holding in an ultrasonic bath five times for 2 seconds each, probes were centrifuged for 10 minutes at 25,000 g at 4°C. The supernatant was used for the following protein quantification, which was carried out using the BCA test [31]. Subsequently, proteins were separated using 10% and 16% SDS-polyacrylamide gel electrophoresis. 20 μg of each sample were denatured together with 5μl application buffer at 70°C for 10 minutes before applying it to the gel. After pipetting into the pockets of the stacking gel, the gel run at 110 V for 1.5 hours. The separated proteins were blotted to PVDF (polyvinylfluorid) membranes (Bio-Rad, Feldkirchen, Germany). The blots were blocked in 3% bovine serum albumin (BSA) or milk powder for 1 h. Incubation with the specific primary antibody, Anti-Surfactant Protein B (Cat # 48604, Seven hills, Cincinnati, USA, 1:1000) diluted in 3% BSA and Anti-Surfactant Protein A (Cat # 31-1221-00, RevMab Bioscience, Burlingame, USA, 1:1000), diluted in milk powder overnight at 4°C followed. After several times of washing in Tris-buffered saline with 0.1 Tween-20 (TBST), the membranes were incubated with the secondary antibody (goat anti-rabbit- IgG, Cat # 111-036-144, Dianova, Hamburg, Germany, 1:20 000) for 1h. The protein-antibody complex was detected with enhanced chemiluminescence (ECL 500 solution A, Amersham Bioscience, Freiburg, Germany). For each SP two western blots were carried out and analyzed. Each electrophorese gel contained 15 gel bags. Therefore, it was possible to load one gel with 15 different protein samples. Each gel contained one loading control, 3 different lung samples per age group with exception of samples of adult lungs, here 2 samples were loaded. Doing so, SP were proofed in 6 different lung samples of 3, 7, 14 and 21 days old rats and 4 different lung samples of adult rats.

Distinct gel bands were detected using the ChemiDoc Touch System (BioRad Laboratories, Feldkirchen, Germany) and quantified by densitometric determination with the Image QuantTM (Amersham Biosciences). Data were normalized to α-actin using a normalization factor, the loading control for homogenates (α-Aktin, Cat # cs-32251, Santa Cruz, Dallas USA, 1:1000).

#### Real time quantitative (RT-q)-PCR analysis

RNA isolation from frozen lung samples was carried out using the NucleoSpin KIT system from Marchery Nagel (Fisher scientific GmbH Schwerte, Germany) following the manufacturers guidelines. Within the KIT there is one step including the treatment with DNAse, so that there cannot be a contamination with gDNA. For reverse transcription of RNA into cDNA, 1μg of the RNA of all samples was incubated with 15μl water, 4μl of 5xiScript Mix and 1μl of iScript reverse transcriptase (BioRad Laboratories, Feldkirchen, Germany) in the thermal cycler for 5 minutes at 25°C, 20 minutes at 46°C and 1 minute at 95°C. After the run, the samples were cooled down to 4°C until they were removed from the device and the cDNA was then stored at -20°C. For the RT-q-PCR, samples were evaluated in triplicate. ß-actin was used as housekeeping gene. The primers used for the SP-A and SP-B gene are listed in Table 1. The primers were diluted to 300 nM by adding 5 μL of reverse and forward to 190 μL of water for the primer mix. 0.8 μL of cDNA and 9.2 μL of master mix (iTaq Universal SYBR Green Supermix, Cat # 1725120, BioRad Laboratories, Feldkirchen, Germany) were added per well. Subsequently, the plate was briefly centrifuged and then analyzed in the ThermoCycler (Bio-Rad, Feldkirchen, Germany). After a 2-minute start at 95°C, the program includes 39 cycles of 5 seconds 95°C and 20 seconds 60°C. Finally, the temperature was increased from 65°C to 95°C in 0.5°C increments. Results were expressed as mean ΔCq values normalized to β-Actin or as ΔΔCq values related to adults regarded then as controls.

**Table 1:** Primer for Rtq PCR.

	**Sequenzen**
**ß-Actin forward** **reverse** **SP-A forward** **reverse** **SP-B forward** **reverse**	ATCCTCTTCCTCCCTGGAGA AGGATTCCATACCCAAGAAGGA CCTGCAGGCTCTGTATGTGG TGCACTTGATACCAGCGACA ACACAGGACCTCTCTGAGCA CCAGCACACCCTTGGGAATC

### Immunohistochemistry

Lungs were instilled with a mixture of cryo-gel Tissue Tec (OCT, Torrance, CA, USA) and PBS (1:3) using a special instillation device as described earlier [15]. After full inflation, the lungs were slowly frozen on dry ice and stored in aluminum foil at -80° C for further processing. Using a cryostat (Leica CM 30505, München, Germany), 6 μm thick sections of the lung were cut based on an age-appropriate section protocol. For evaluation, sections of different section planes were chosen randomly. The immunohistochemistry was performed with a substrate staining method. The sections were first blocked with 3% peroxidase for 5 minutes in the dark and after brief washing with distilled water incubated with 10% normal serum/goat (Dako, Glostrup, Denmark) for 20 minutes. The sections were then blocked with avidin and biotin (Vector Labratories, Newark, USA) for 15 minutes each. Afterwards, the primary antibody rabbit anti SP-A (Revmab Bioscience, Cat # 31-1221-00, Burlingame, USA, 1:100) and rabbit anti SP-B (Merck, Cat # ABS21Dasmstadt, Germany, 1:100) was added to the sample in Tris buffered saline and incubated overnight at 4°C. The next morning, the samples were rinsed again and incubated for one hour with the secondary antibody (goat anti rabbit biotinylated, Cat # E0432, Dako, Glostrup, Denmark, 1:1000) at room temperature. After rinsing again, an incubation with the ABC Kit Peroxidase (Vector Labratories, Newark, USA) followed. The staining was performed with the DAB peroxidase substrate (Vector Labratories, Newark, USA) for 10 minutes. Subsequently, for nuclear staining, the sections were stained with hematoxilin for 45 seconds before the analysis followed.

### Perfusion fixation

For determining the lung maturation stereologically, a cannula was connected with a hydrostatic pressure column. Both lungs were flushed with NaCl/heparin solution for 10 min via the right ventricle, through incision of the left atrium. At the end of perfusion, the lungs were inflated up to 30 cm H_2_O, deflated to 10 cm H_2_O and then fixed by vascular perfusion with 1.5% paraformaldehyde and 1.5% glutaraldehyde in 0.15M HEPES buffer. Afterward the heart-lung block was removed and fixed by immersion for at least 24h. Before sampling and further processing the lung volume was determined by the Archimedes principle [32]. Afterwards lungs were embedded in 2% aqueous agar, and each organ was cut from apical to caudal into 2 mm slices using a tissue slicer as described in more detail earlier [33]. Tissue slices were taken starting alternately with a random number. Embedding followed completely in glycol methacrylate (Technovit 8100) or in epoxy resin, after further subsampling, into small tissue blocks as already described [16]. 1.5 μm methylacrylate and 1 μm epon sections were cut and stained with toluidine blue.

### Stereology

The stereological evaluation was carried out using the point and intersection counting according to the guidelines for quantitative assessment of lung structure [34]. Therefore, the software visiopharm (Visiopharm, Hoersholm, Denmark) was used. Test fields were sampled all over the section by the software according to the systematic random sampling to guarantee that each area of the section has the same chance to be evaluated. Doing so, 20% or 30% of one section were evaluated.

After immunohistochemical staining of cryosections, the surface fraction of SP-labelled AEII was evaluated as described earlier [14,15,35,36]. Briefly, the number of intersections with labelled AEII and with the alveolar surface were counted and were given as percentage of the septal surface. The relative surface fraction of SP-labelled AEII (Intersections AEII / AEII + AEI; %) is a suitable tool for quantifying the amount of AEII showing sufficient labelling. Per animal at least 3 samples of different regions of the lungs were evaluated, so that between 100 and 200 counting events per parameter (e.g. intersections with positive cells) could be counted so that more than 100 positive labeled cells per animal were counted.

To get information about morphological lung maturation, the surface (S_V_(septa/par)) and volume densities (V_V_(septa/par) of alveolar septa related to parenchyma, the volume densities of distal airways (V_V_(airsp/par)) related to parenchyma, as well as the mean barrier thickness (τ), were determined as described elsewhere [15,16,37]. To get values independent of the reference space surface and volume densities were multiplied with V_V_(par/par + nonpar) and lung volume [37].

## Statistics

All values are given in mean ± SD. Significant differences were evaluated using the One-Way-ANOVA test for normally distributed values and the nonparametric Kruskal-Wallis test for not normally distributed values. Multiple comparisons were corrected with the Dunnett´s multiple comparison test. Correlation analyses were carried out using the non-parametric Speaman correlation test. A level of two tailed p< 0.05 was considered to be significant. The Graph Pad Prism 6.07 (Statcom, Witzenhausen, Germany) was used.

## Results

### Histology of lung parenchyma

**Fig 1:**
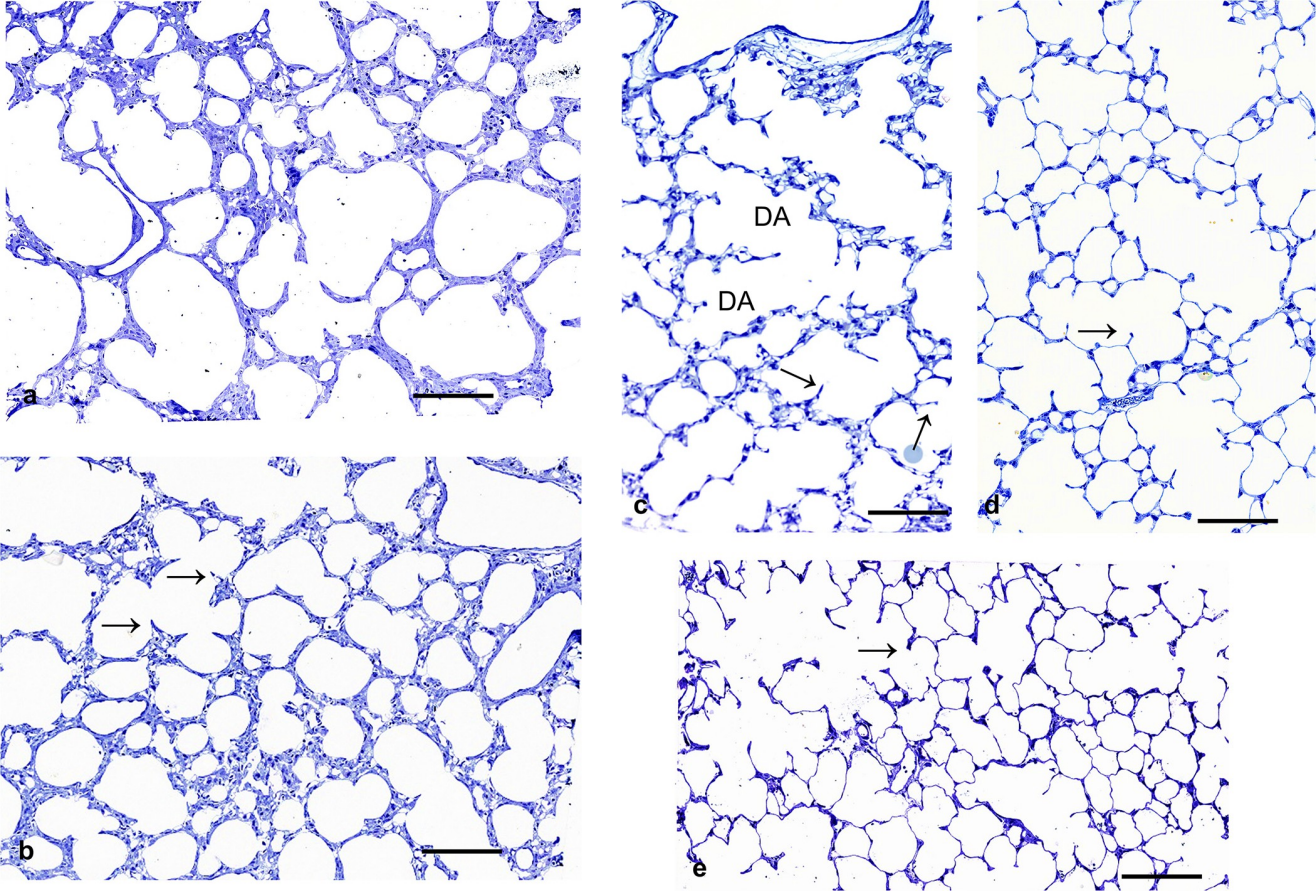
Histology of lung parenchyma. a) 3 days old rats: more or less pronounced saccules surrounded by thick primary septa containing a double layered capillary bed are visible (scale bar; 200μm). b) 7 days old rats: alveolarization has started. Protrusions of secondary septa are seen sporadically (arrows). The septa appear still thick (scale bar; 200μm). c) 14 days old rats. Alveolarization is almost completed. Ductus alveolares (DA) are visible. Protrusions of secondary septa are seen frequently. The size of air spaces has been decreased (scale bar; 200μm). d) 21 days old rats: numerous alveoli with more or less small alveolar septa are seen. The capillary bed is single layered. The size of airspaces is smaller than in the younger developmental stages (scale bar; 200μm). e) Adult rats with thin alveolar septa and numerous small alveoli (scale bar; 200μm).

3 days (d) old pups showed no signs of alveolarization. More or less wide sacculi are visible. The so called primary alveolar septa equipped with double capillary beds look remarkable thick (Fig 1A). Lungs of 1 week old rats showed signs of alveolarization. Sprouts of secondary septa from the primary septa are visible (Fig 1B). The septa are moderately thick, partly some remarkable thick septa are visible. They still contain a double capillary bed. The terminal airspaces seem to get smaller. Ductus alveolares and sacculi could not be clearly differentiated. In lungs of 14 days old rats alveolarization has proceeded and partly numerous alveoli lined with secondary septa are visible. Ductus alveolares and alveoli can be differentiated (Fig 1C). The alveolar septa containing primarily two capillaries are much smaller than in 7 days old lungs. After 21 postnatal days, secondary septa with mainly single layered capillary beds are seen (Fig 1D). In adults, lung parenchyma consists of many alveoli and ductus alveolares (Fig 1E).

### Stereological parameters characterizing alveolarization

**Fig 2:**
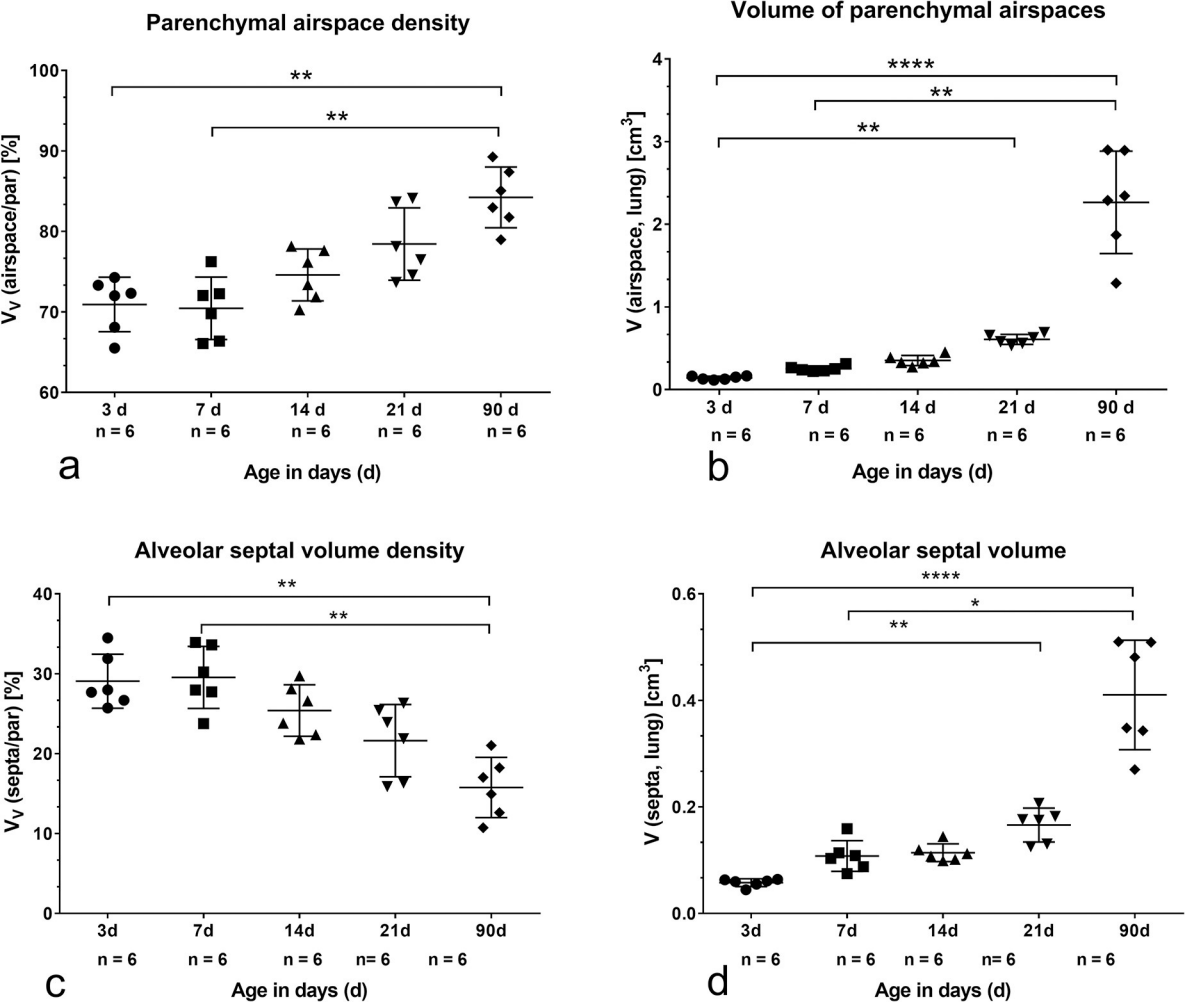
Stereological parameters: Volume density and total volume of parenchymal airspace and alveolar septa during postnatal development. P values: *p<0.05, **p<0.01, ****p<0.0001. a) V_V_ (airspace/parenchyma). b) Total volume of parenchymal airspace related to right lung (airspace, lung). c) V_V_ (septa/parenchyma). d) Total volume of alveolar septa related to right lung (septa, lung).

During alveolarization the volume fraction as well as the total volume of parenchymal airspaces did not differ (Fig 2A and 2B). Compared to 3 d old rats there was a significant increase of parenchymal airspace volume at the end of morphological maturation (Fig 2B). The septal volume fraction did not change significantly during bulk alveolarization (Fig 2C). In adults, the septal volume fraction was significantly lower compared to 3 and 7 d old rats (Fig 2C). However, the septal volume was already at the end of morphological lung maturation significantly higher compared to 3 d old rats (Fig 2D).

**Fig 3:**
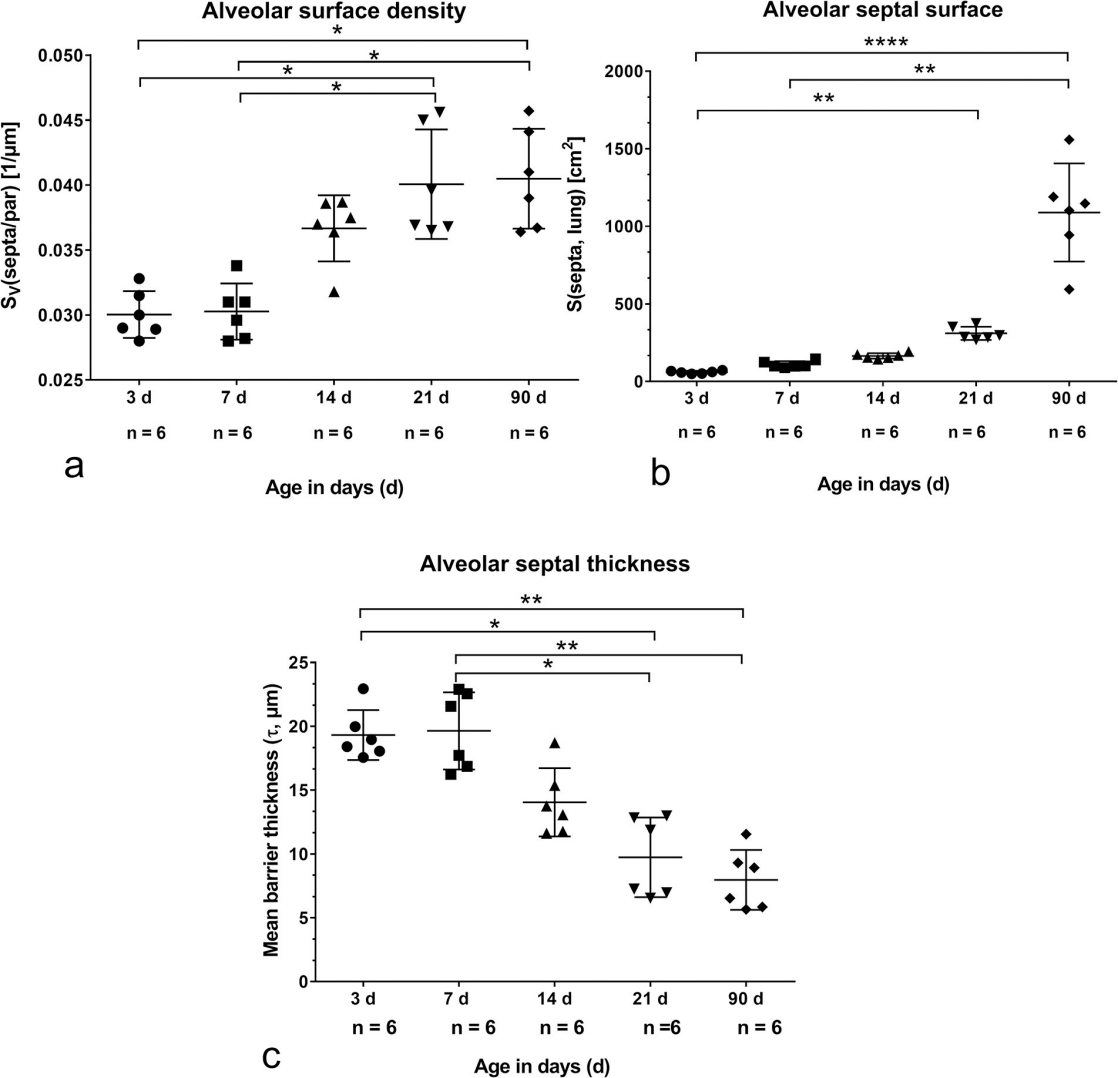
Stereological parameters: Surface density and total surface of alveolar septa as well as septal thickness during postnatal development. P values: *p<0.05, **p<0.01, ****p<0.0001. a) S_V_ (septa/parenchyma). b) Total surface of alveolar septa related to right lung (septa, lung). c) Thickness of septa presented as mean barrier thickness.

The surface density of the septa as well as the total septal surface differed on postnatal day 21 significantly compared to postnatal day 3 (Fig 3A and 3B). The significant increase of septal surface can be regarded as indirect hint for alveolarization. 21 days after birth the septal thickness was significantly lower compared to postnatal day 3 (Fig 3C). Between the end of morphological maturation and the adult age no differences in septal thickness were visible.

Thus, the end of morphological maturation on postnatal day 21 was characterized by significantly increase in volume of parenchymal airspaces, alveolar septa as well as in surface of septa and decrease in septal thickness. After the end of morphological maturation on day 21, parameters characterizing morphological lung maturation showed no differences compared to adult lungs (Figs 2 and 3).

### SP-A labeling

**Fig 4:**
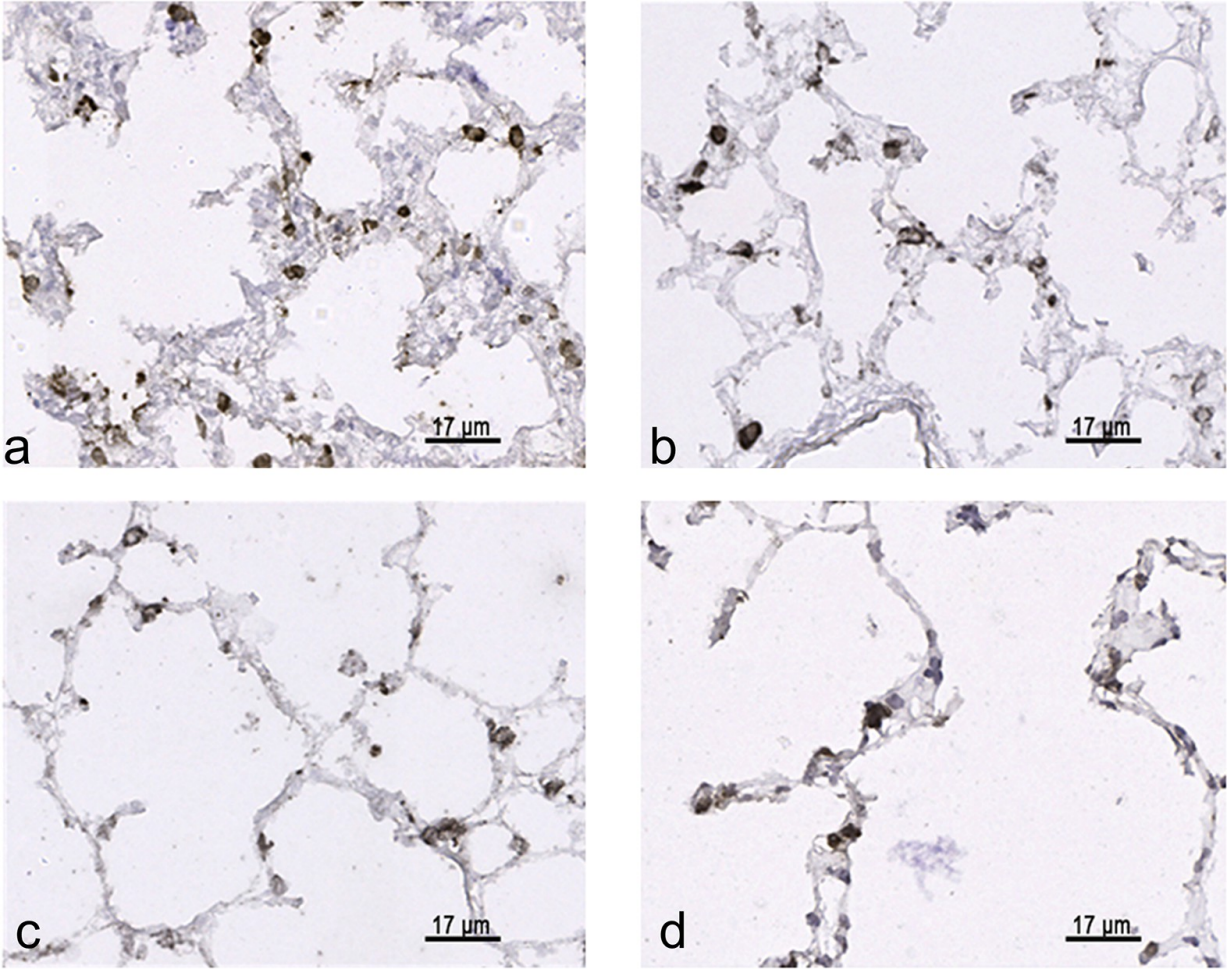
SP-A labeling of alveolar epithelial cells type II (AEII). Immunolabeling of cryostat sections from 3-day-old (a), 14-day-old (b), 21-day-old (c), and adult (d) rats. Lung parenchyma exhibits SP-A positive AE II.

Lung parenchyma of newborn 3 days old pups showed numerous SP-A labeled AEII distributed more or less in corners of thick alveolar septa with a double-layered capillary bed (Fig 4A). After 14 days during the so-called bulk alveolarization, the alveolar septa were smaller, the capillary bed remained mostly double layered and the SP-A labeling of AEII seemed less numerous (Fig 4B). At the end of morphological lung maturation (21 d old rats) the capillary bed was single layered, the septal thickness was comparable with adults and the SP-A labeling showed no clearly visible alterations compared to adult lungs (Fig 4C and 4D).

**Fig 5:**
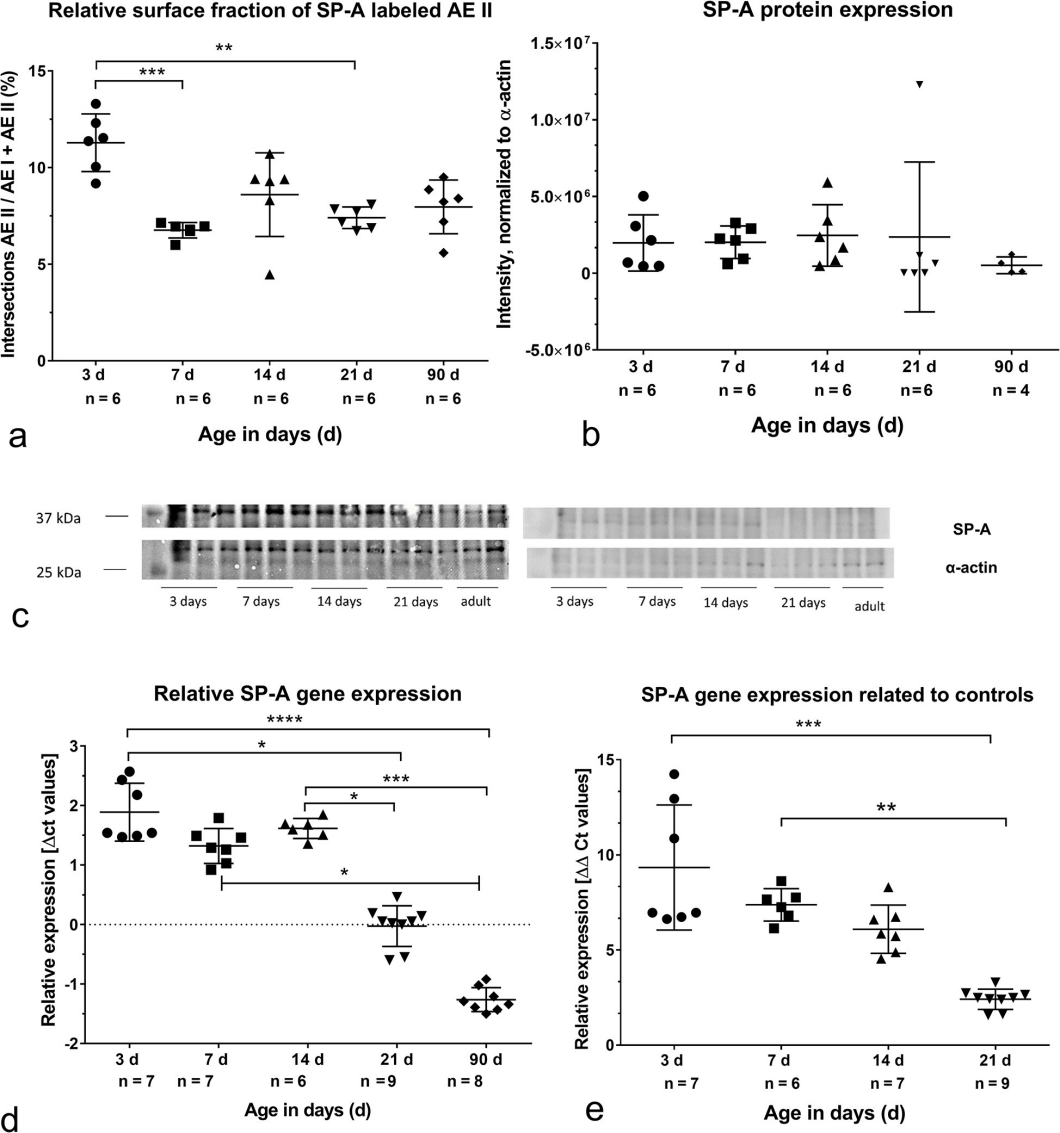
Expression of surfactant protein A (SP-A): Immunohistochemical level, protein expression level, gene expression level. P values: *p<0.05, **p<0.01, ***p<0.001. a) Relative surface fraction of SP-A labeled alveolar epithelial cells type II (AEII). b) SP-A protein expression (western blotting). c) Western blots used for densitometry. d) Relative SP-A gene expression (mRNA content). e) SP-A gene expression related to controls.

Determining the relative surface fraction of SP-A labeled AEII, a significant decrease during alveolarization and at the end of morphological lung maturation was evaluated (Fig 5A). However, the protein expression of SP-A on postnatal day 3 was comparable with values during and after alveolarization (Fig 5B). The protein expression values based on densitometric determination of 2 western blots (Fig 5C). The relative expression of the SP-A gene showed the significant highest levels before alveolarization. At the end of morphological lung maturation, the gene expression was significantly reduced. The significant lowest values were seen in lungs of adults (Fig 5D). If the expressions were related to the adult animals as a comparison group and the expressions of the adult animals are set as 100% (ΔΔct values), the AEII of the 3 and 7 days old lungs showed significantly higher values with 7 to 8 times the expression compared to the 21 days old animals (Fig 5E).

### SP-B labeling

**Fig 6:**
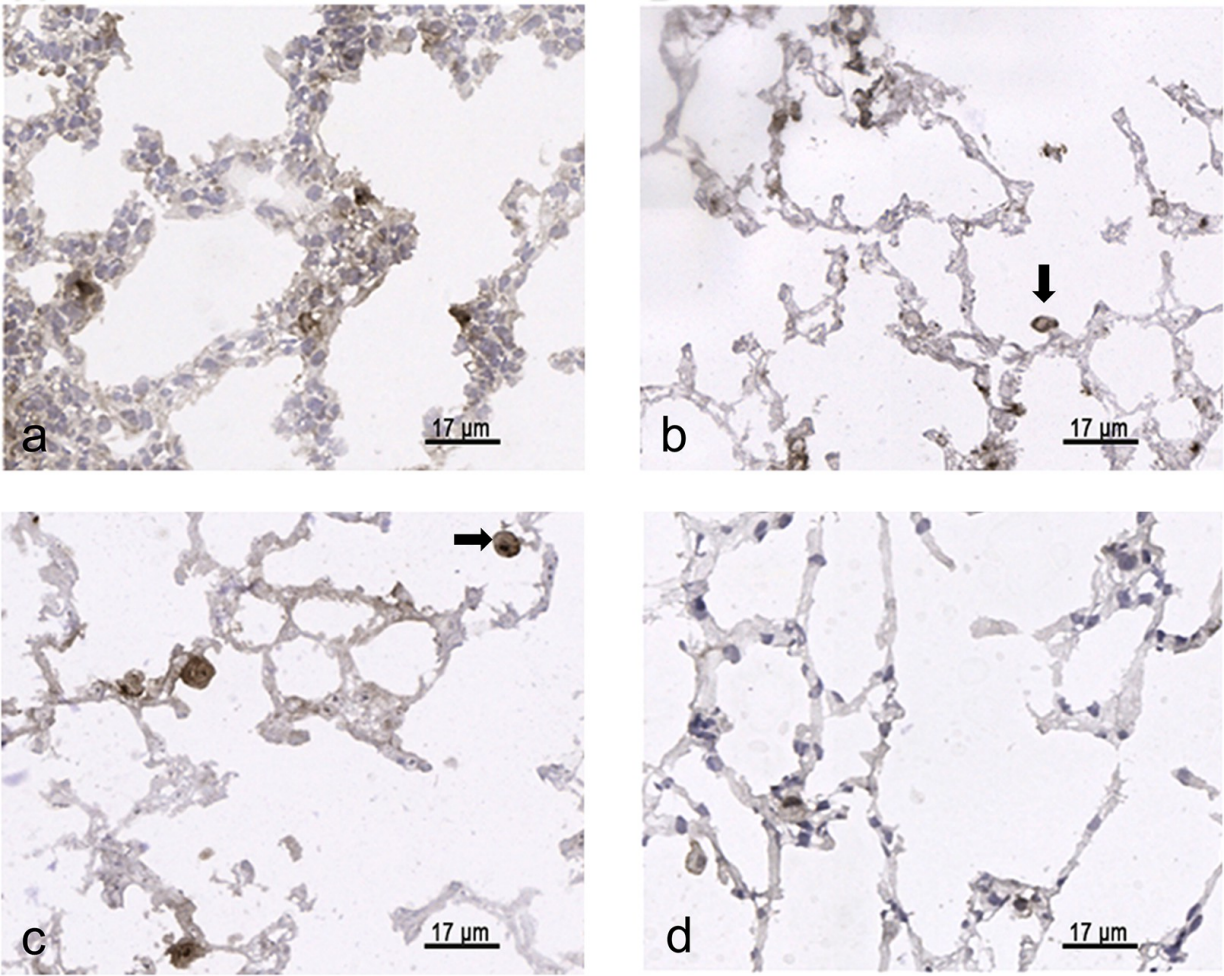
SP-B labeling of alveolar epithelial cells type II (AEII). Immunolabeling of cryostat sections from 3-day-old (a), 14-day-old (b), 21-day-old (c), and adult (d) rats. Lung parenchyma exhibits SP-B positive AEII.

**Fig 7:**
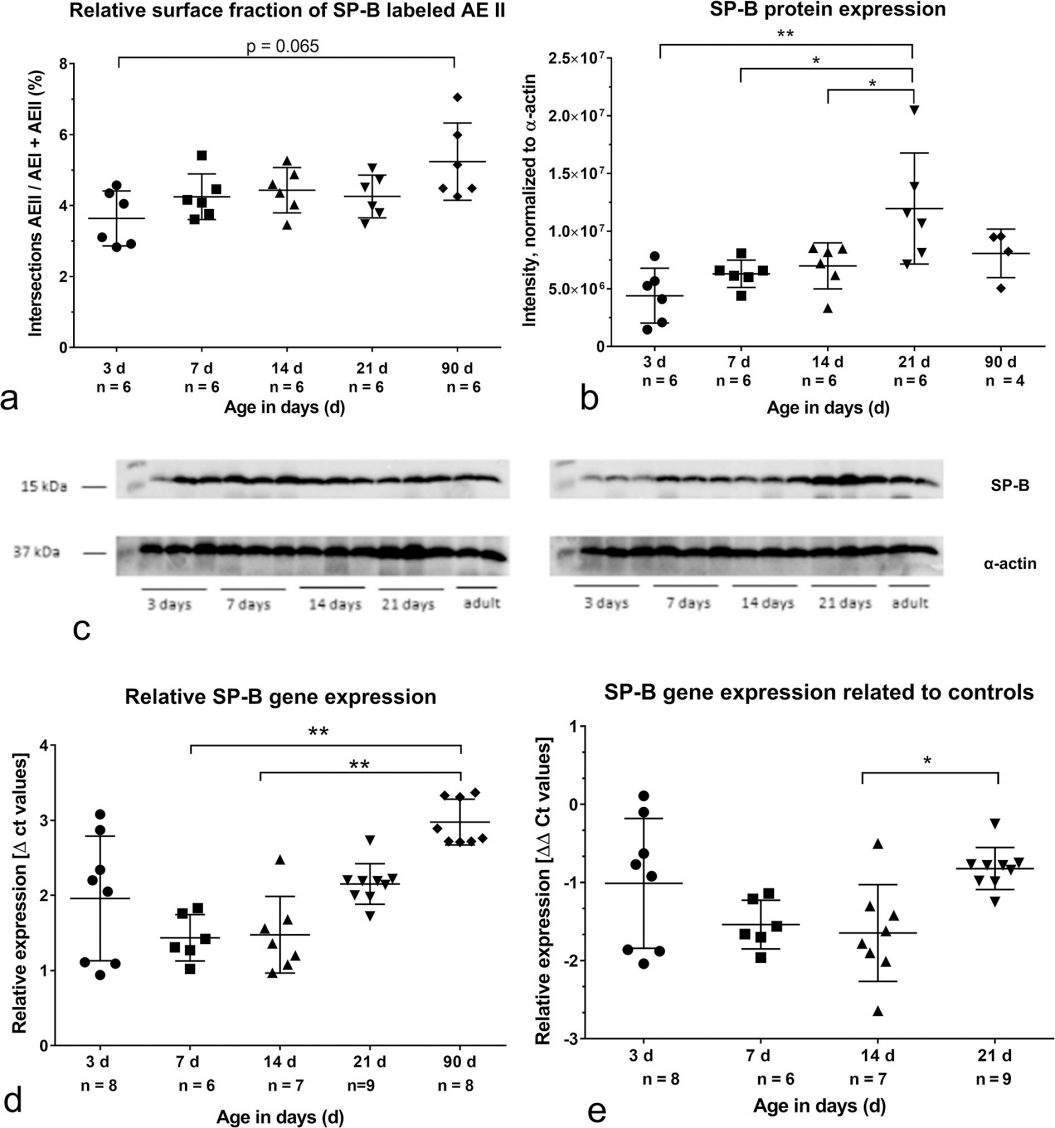
Expression of surfactant protein B (SP-B): Immunohistochemical level, protein expression level, gene expression level. P values: *p<0.05, **p<0.01. a) Relative surface fraction of SP-B labeled alveolar epithelial cells type II (AEII). b) SP-B protein expression (western blotting). c) Western blots used for densitometry. d) Relative SP-B gene expression (mRNA content). e) SP-B gene expression related to controls.

Before alveolarization, the thick septa surrounding saccular airspaces contain some SP-B labeled AE II (Fig 6A). Qualitatively no differences of SP-B labeling were found during morphological lung maturation. Labeling was comparable with that of 3 days old lungs after 14 and 21 days (Fig 6B and 6C). In the alveolar space SP-B labeled alveolar macrophages were visible (Fig 6B and 6C, arrows). The SP-B labeling of AEII in the corners or within the thin septa in lungs of adult rats did qualitatively not differ from the other groups (Fig 6D). However, determining the relative surface fraction of labeled SP-B, a significant increase of SP-B labeled AEII in adult lungs compared to newborn was observed (Fig 7A).

Looking at the protein expression, no differences of the labeling intensity were found before and after alveolarization. The significant highest protein expression was determined at the end of morphological lung maturation compared to values obtained before alveolarization occurred (Fig 7B). The protein expression values based on densitometric determination of 2 western blots (Fig 7C).

The relative gene expression of SP-B showed comparable values during alveolarization. The highest values were seen in lungs of adult rats. Significant differences were determined between 7, respectively 14 days old lungs compared to adults (Fig 7D).

The SP-B expression values normalized to the housekeeping gene and to the control group do not show any alterations before and during alveolarization but significantly higher values at the end of morphological maturation at postnatal day 21 (Fig 7E).

## Correlation between SP-A expression and septal surface

To get more information about a possible relationship between morphological lung maturation and surfactant maturation during postnatal lung development in rats, we carried out some correlation analyses between the expression of the investigated surfactant proteins and the surface of alveolar septa. The increase of septal surface is caused by formation of new alveolar septa and is therefore an indirect hint for alveolarization.

**Fig 8:**
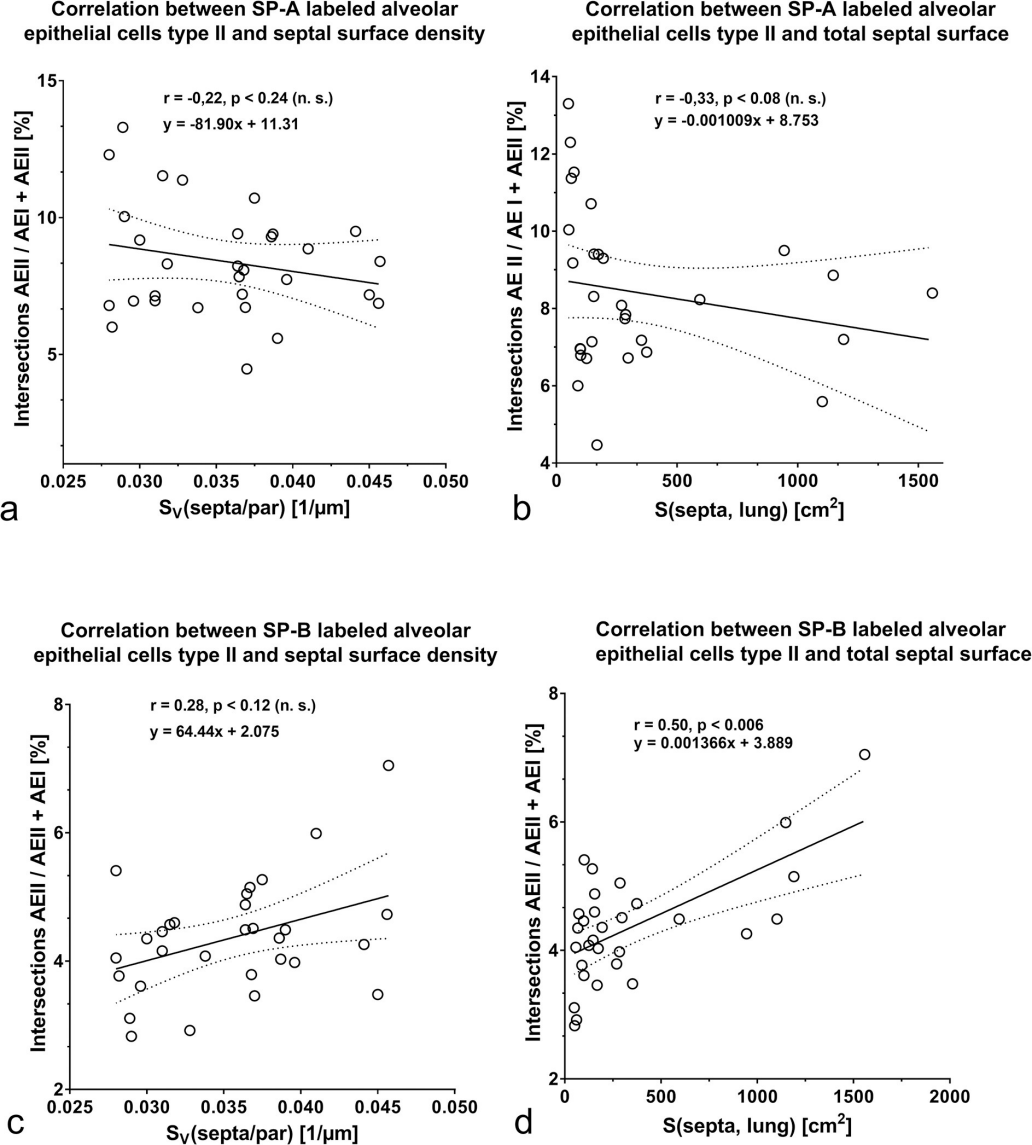
Correlation analyses between a) relative surface fraction of SP-A labeled AEII and septal surface density. b) relative surface fraction of SP-A labeled AEII and total septal surface. c) relative surface fraction of SP-B labeled AEII and septal surface density. d) relative surface fraction of SP-B labeled AEII and total septal surface.

**Fig 9:**
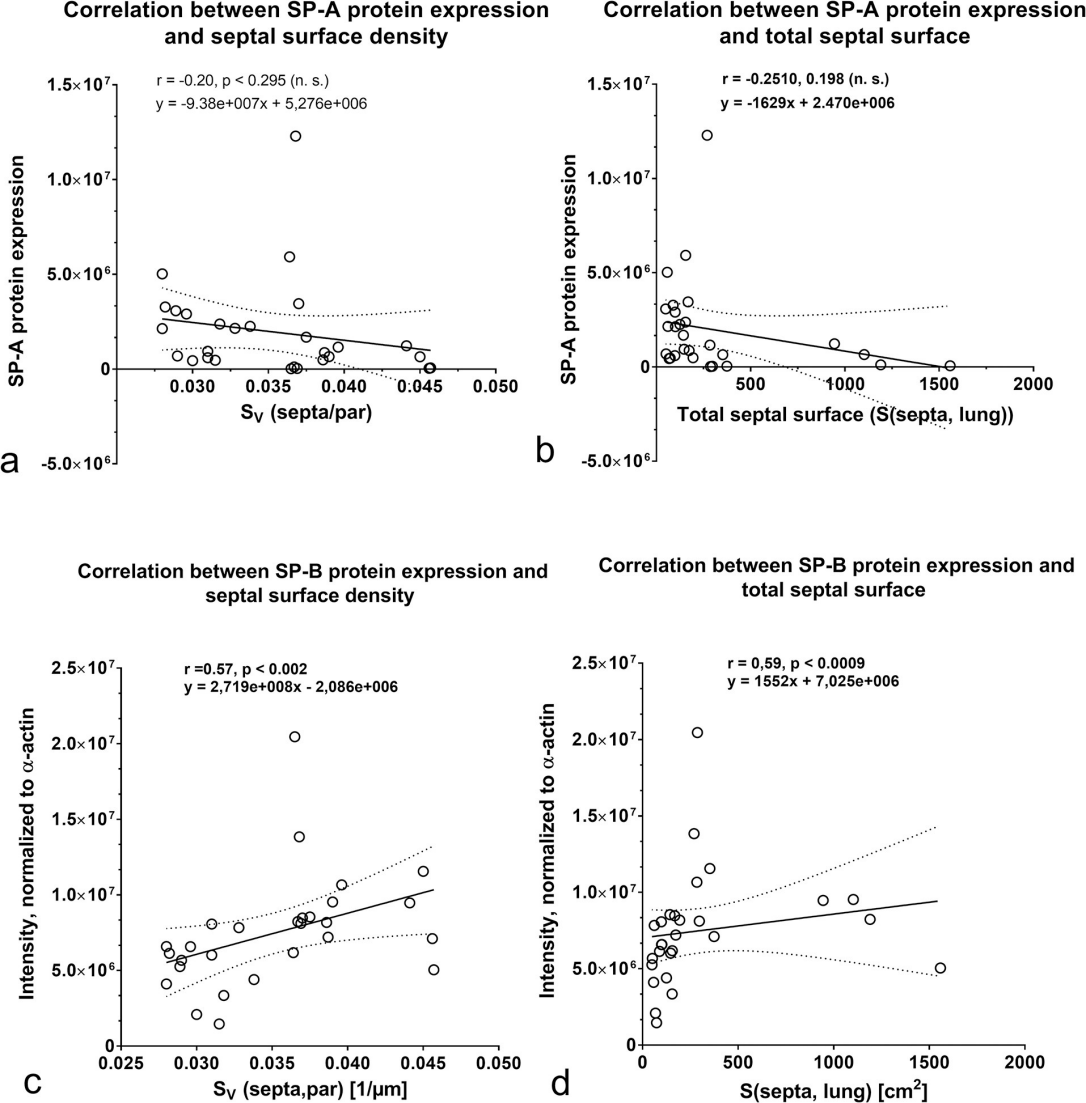
Correlation analyses between a) SP-A protein expression and septal surface density. b) SP-A protein expression and total septal surface. c) SP-B protein expression and septal surface density. d) SP-B protein expression and total septal surface.

**Fig 10:**
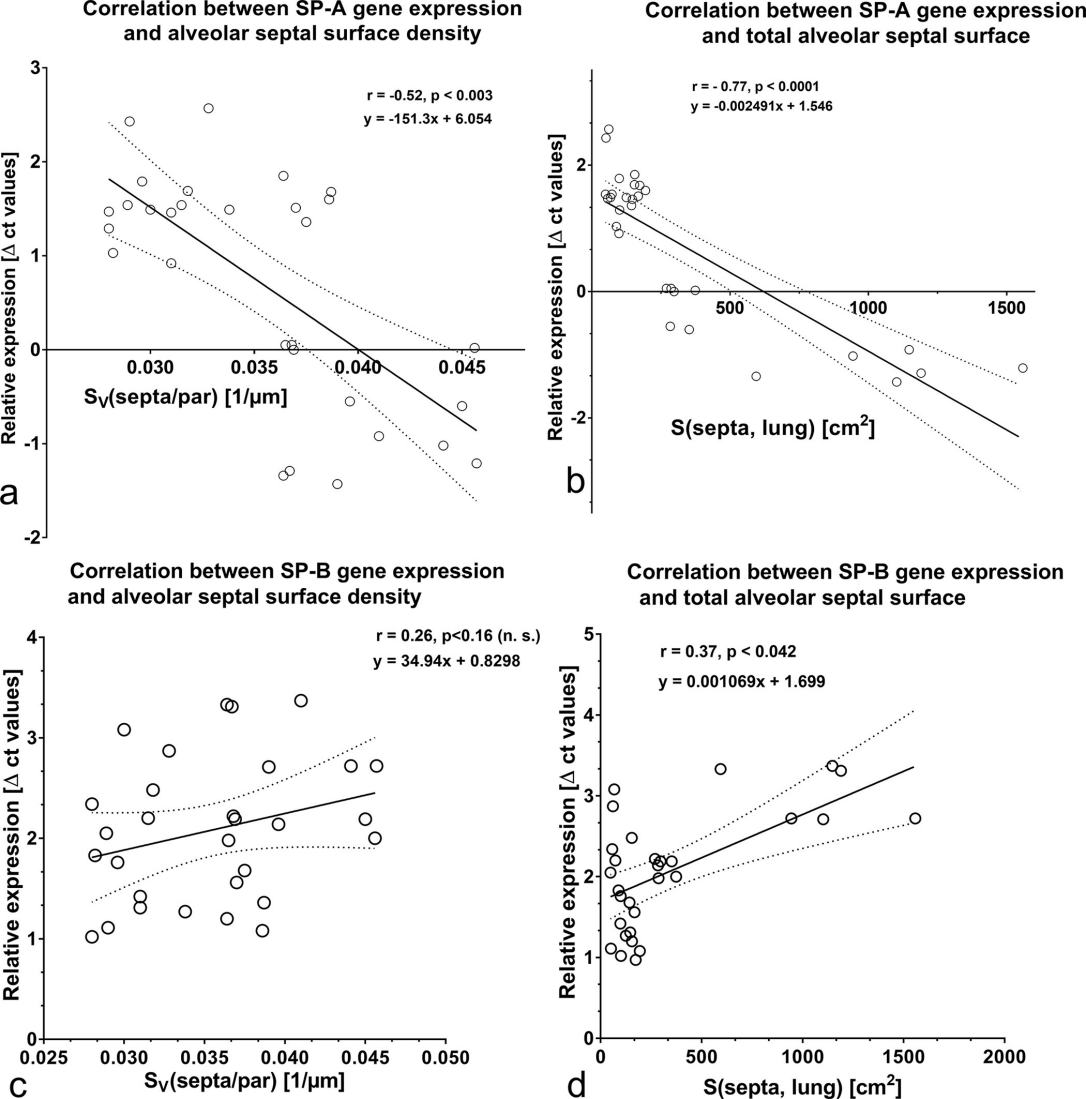
Correlation analyses between a) SP-A gene expression and septal surface density. b) SP-A gene expression and total septal surface. c) SP-B gene expression and septal surface density. d) SP-B gene expression and total septal surface.

Regarding the SP-A expression, no relation was found between the percentage of SP-A labeled AE II and the increase of alveolar septal surface (Fig 8A and 8B). The SP-A protein content exhibited no relation with total surface of alveolar septa (Fig 9B). A significant negative correlation was found between SP-A gene expression and the formation of alveolar septa (Fig 10A and 10B). Looking at the expression of SP-B, we ascertained a positive correlation between increase of the percentage of SP-B labeled AE II and total septal surface (Fig 8D). A positive correlation was also determined between the SP-B protein content and the septal surface density as well as the septal surface (Fig 9C and 9D). SP-B gene expression values correlated positively with the total septal surface (Fig 10D).

## Discussion

This study examined the expression of the SP-A and SP-B during postnatal morphological lung development in rats. On the one hand, our results showed clearly that on day 21 stereological parameters characterizing morphological lung maturation, are comparable to those of adults, but significantly differ compared to values of newborn lungs before alveolarization. The increase of total septal surface combined with the decrease of septal thickness is regarded as an indirect hint for the increase of the number of terminal gas-exchange units by secondary septation out from the walls of saccules and therefore an indirect hint for alveolarization. These results are in accordance with other publications, but differ with regard of time points, species and question of the study [10,11,14,16,38–41]. An increase of the number of alveoli within this time period was additionally shown by others in rat and mouse lungs [16,41]. On the other hand, the investigated surfactant proteins exhibit partly different patterns of expression independent of the stages of morphological lung maturation. In detail, the relative surface fraction of SP-A labeled AEII and the gene expression of SP-A showed the highest values in the first days after birth, before alveolarization started and decreased significantly until the end of morphological lung maturation. Our results are in accordance with those of other studies. Using the enzyme-linked immunosorbent Assay (ELISA), an increase in the SP-A content with a maximum on the first postnatal day was determined [42–44]. Also the content of alveolar and total lung SP-A relative to body weight and surface area was higher on the first postnatal day than in all other age groups [45]. The very high gene expression values of SP-A, and the relative surface fraction of SP-A labeled AEII may be explained by its dual function [46]. SP-A as well as SP-D have an immunomodulating effect [43,47]. SP-A and SP-D belong to a protein family called collectins [48]. The lectin domain binds on the surface of pathogens to opsonize them for uptake by phagocytes [48]. The collectins also have the ability to modulate the functions of cells of the adaptive immune network such as dendritic cells and T lymphocytes [48,49]. This immunological function is particularly important in the first few days after birth, as the animals get in contact with many different pathogens. The SP´s support the innate immune defense, since the adaptive immune defense in newborns is not yet developed and has to develop first [50], which explains the higher surface proportion of the SP-A marked AEII. The expression rate of SP-A in newborn lungs is probably highest due to the first exposure to various foreign substances in the inhaled air associated with birth. Some investigations support this assumption. So lower concentrations of SP-A in prematurely born children with an immature surfactant system (< 32 weeks) [42] are associated with an increased risk of infection. Studies on SP-A knockout mice also showed an increased susceptibility to infection [51,52]. Surprisingly, the measured SP-A protein expression showed no differences and great variation before, during and after morphological lung maturation. One explanation may be that lungs were not lavaged. Therefore, the SP-A tissue content in the tissue blocks had different origin. It comes not only from AEII, but also from club cells of the bronchiolar or bronchial epithelium, which also synthesize SP-A [22,36,53–55]. The tissue also comprised intra-alveolar SP-A, bound to tubular myelin or to the surface film [56,57]. Additionally SP-A was taken up by alveolar macrophages [54,58]. The superposition of SP-A synthesized in AEII or cube cells or reabsorbed by macrophages and intra-alveolar SP-A bound to phospholipids in the tissue blocks of the not lavaged lungs may be one reason for the constant protein levels independent of the postnatal age. However, correlation analyses of SP-A expression and alveolarization independent of the age, a not significant interrelation between alveolar septal surface and SP-A expression was found. Our results indicate that the gene expression of SP-A and the alteration of alveolar septal surface are linearly related to each other. SP-A expression decreased with increased septal surface area.

The expression rate of SP-B during postnatal development differs considerably in comparison to SP-A. Before and during the formation of secondary septa, the surface fraction of SP-B labeled AE II, the SP-B protein expression as well as the m-RNA content exhibits no differences. At the end of remodeling and maturation of interalveolar septa as well as of the capillary bed, the SP-B protein expression increases, whereas the surface fraction of SP-B labeled AEII and the mRNA content reached significant higher values in adults. The data show also quite well that before and in the alveolarization phase SP-B is continuously transcribed, translated and, after post-translational modification, stored in multivesicular bodies, in lamellar bodies and thus becomes immunohistochemically detectable without any significant differences. The significant increase in tissue protein expression of SP-B after the end of morphological lung development possibly indicates an overlay with SP-B synthesized in the club cells and/or taken up by macrophages [54,59,60]. Moreover, in the not lavaged lungs intracellular SP-B as well as intra-alveolar SP-B was determined. Because the significant increase of SP-B protein content at day 21 is linked with the end of bulk alveolarization leading to a strong enlargement of the septal surface. Thus, increased amounts of SP-B are necessary for the formation of the phospholipid film in the alveolar hypophase of the numerous new formed alveoli may be also conceivable. Looking at the mRNA content, differences are visible between adults, 7 and 14 days old lungs. No differences are seen between 21 days old lungs and adult lungs. The SP-B labeling of AEII is firstly significant in adults. Summarizing this, it becomes clear, that the level of SP-B expression and the maturity level e.g. alveolarization are related to each other, which is also proofed by correlation analyses, showing a linear relationship between SP-B expression and alveolarization. Thus, SP-B expression increases with the increase of septal surface to guarantee a continuous spreading of surfactant over the increasing inner alveolar surface, because SP-B is responsible for the spreading of the surfactant film [29]. Therefore, in adults, which finished the bulk and continued alveolarization phase, the significant highest septal surface is combined with the significant highest mRNA content and surface fraction of SP-B labeled AEII. At this point of time, the alveoli are fully developed, so that an increased production of SP-B is necessary and accordingly an increased gene expression of SP-B is to be expected for the stabilization of the alveoli. While the expression rates of SP-B are still comparable before, during and after bulk alveolarization, significantly increased values are found in adult age compared to the times when alveolar formation takes place more intensively. This suggests that in adult animals the reduction in surface tension is accompanied by increased stabilization of the phospholipids and that accordingly the gene expression rate of SP-B has to adapt to the increased requirements.

Our results regarding the expression of SP-B genes partly correspond to results of Randell et al., who detected an increased tissue content of SP-B mRNAs before birth, a decrease during the first postnatal week, and then again an increase reaching the highest levels in adults [22].

Comparative studies of morphological and functional lung maturation are not yet available. This is to our knowledge the first study, which determines the expression of the two crucial SP on the histological, protein and RNA level at different times during postnatal lung development in rats and its relation to stereologically determined parameters of lung development.

However, one limitation of our study is that we did not carry out a differential analysis with respect on sex. There exist several studies indicating a critical role of sex hormones in fetal lung development [61,62]. Female estrogen and progesteron receptor expression peaked prenatal and showed reduced levels at birth and in adult mice [62]. It was found that in fetal rat and rabbit estrogens stimulate both the formation and secretion of surface-active phospholipids and stimulate expression of surfactant protein A and B mRNAs as well as increase the number of AEII and the formation of lamellar bodies [61]. A comparison of prenatal lung development of female and male mouse lungs based on interacting genes, surfactant related genes and several genes of regulating lung development, showed a clearly delay in prenatal lung maturation of male in the canalicular period [63]. The prenatal transition from the canalicular to the saccular period is characterized by a surge of surfactant production. Data about remaining delay in lung development and maturation after birth are missing. However, a study comparing female piglets and male piglets delivered one day before birth by caesarian section, showed lower SP-B and VEGF expressions in their lungs, but comparable alveolarization was measured by alveolar counts [64]. Further studies are necessary to proof whether the described estrogen dependent prenatal acceleration of lung maturation primarily in the late fetal lung development continues postnatally.

## Supporting information

S1 File Stereological data. (PDF)

S2 File SP surface fraction, western blot data, (RT-q)-PCR q data. (PDF)

S3 File Correlation analyses data for Fig 8. (PDF)

S4 File Correlation analyses data for Fig 9. (PDF)

S5 File Correlation analyses data for Fig 10. (PDF)

## References

[1] Agudelo CW, Samaha G, Garcia-Arcos I (2020) Alveolar lipids in pulmonary disease. A review. Lipids Health Dis 19: 122. doi: 10.1186/s12944-020-01278-8 [pii]. 32493486 PMC7268969

[2] Bland RD (2005) Neonatal chronic lung disease in the post-surfactant era. Biol Neonate 88: 181–191. 87581 [pii]; doi: 10.1159/000087581 16210840

[3] Schittny JC (2017) Development of the lung. Cell Tissue Res 367: 427–444. doi: 10.1007/s00441-016-2545-0 [pii]. 28144783 PMC5320013

[4] Duncker H (1990) Respirationstrakt. In: von Hinrichsen K, editors. Humanembryologie. Heidelberg: Springer. pp. 571–606.

[5] Berger J, Bhandari V (2014) Animal models of bronchopulmonary dysplasia. The term mouse models. Am J Physiol Lung Cell Mol Physiol 307: L936–L947. ajplung.00159.2014 [pii]; doi: 10.1152/ajplung.00159.2014 25305249 PMC4269689

[6] D’Angio CT, Ryan RM (2014) Animal models of bronchopulmonary dysplasia. The preterm and term rabbit models. Am J Physiol Lung Cell Mol Physiol 307: L959–L969. ajplung.00228.2014 [pii]; doi: 10.1152/ajplung.00228.2014 25326582

[7] Jobe AH (2015) Animal Models, Learning Lessons to Prevent and Treat Neonatal Chronic Lung Disease. Front Med (Lausanne) 2: 49. doi: 10.3389/fmed.2015.00049 26301222 PMC4528292

[8] Nardiello C, Mizikova I, Morty RE (2017) Looking ahead: where to next for animal models of bronchopulmonary dysplasia? Cell Tissue Res 367: 457–468. doi: 10.1007/s00441-016-2534-3 [pii]. 27917436 PMC5320021

[9] Burri PH (2006) Structural aspects of postnatal lung development—alveolar formation and growth. Biol Neonate 89: 313–322. 92868 [pii]; doi: 10.1159/000092868 16770071

[10] Tschanz SA, Salm LA, Roth-Kleiner M, Barre SF, Burri PH, Schittny JC (2014) Rat lungs show a biphasic formation of new alveoli during postnatal development. J Appl Physiol (1985) 117: 89–95. japplphysiol.01355.2013 [pii]; doi: 10.1152/japplphysiol.01355.2013 24764134

[11] Burri PH, Dbaly J, Weibel ER (1974) The postnatal growth of the rat lung. I. Morphometry. Anat Rec 178: 711–730. doi: 10.1002/ar.1091780405 4592625

[12] Burri PH (1984) Fetal and postnatal development of the lung. Annu Rev Physiol 46: 617–628. doi: 10.1146/annurev.ph.46.030184.003153 6370120

[13] Vidic B, Burri PH (1983) Morphometric analysis of the remodeling of the rat pulmonary epithelium during early postnatal development. Anat Rec 207: 317–324. doi: 10.1002/ar.1092070210 6650864

[14] Hupa KL, Schmiedl A, Pabst R, von HS, Stephan M (2014) Maternal deprivation decelerates postnatal morphological lung development of f344 rats. Anat Rec (Hoboken) 297: 317–326. doi: 10.1002/ar.22848 24357522

[15] Wagener I, Jungen M, von HS, Stephan M, Schmiedl A (2020) Postnatal morphological lung development of wild type and CD26/DPP4 deficient rat pups in dependency of LPS exposure. Ann Anat 229: 151423. S0940-9602(19)30127-X [pii]; doi: 10.1016/j.aanat.2019.151423 31654734

[16] Werner J, Schipke J, Brandenberger C, Schmiedl A, Muhlfeld C (2022) Differential temporal development of alveoli and the alveolar capillary network in the postnatal rat lung. Am J Physiol Lung Cell Mol Physiol 323: L667–L675. doi: 10.1152/ajplung.00273.2022 36283087

[17] Clements JA (1997) Lung surfactant: a personal perspective. Annu Rev Physiol 59: 1–21. doi: 10.1146/annurev.physiol.59.1.1 9074754

[18] Olmeda B, Martinez-Calle M, Perez-Gil J (2017) Pulmonary surfactant metabolism in the alveolar airspace: Biogenesis, extracellular conversions, recycling. Ann Anat 209: 78–92. S0940-9602(16)30173-X [pii]; doi: 10.1016/j.aanat.2016.09.008 27773772

[19] Whitsett JA, Weaver TE (2015) Alveolar development and disease. Am J Respir Cell Mol Biol 53: 1–7. doi: 10.1165/rcmb.2015-0128PS 25932959 PMC4566117

[20] Wright JR (1990) Clearance and recycling of pulmonary surfactant. Am J Physiol 259: L1–12. doi: 10.1152/ajplung.1990.259.2.L1 2200279

[21] Khoor A, Stahlman MT, Gray ME, Whitsett JA (1994) Temporal-spatial distribution of SP-B and SP-C proteins and mRNAs in developing respiratory epithelium of human lung. J Histochem Cytochem 42: 1187–1199. doi: 10.1177/42.9.8064126 8064126

[22] Randell SH, Silbajoris R, Young SL (1991) Ontogeny of rat lung type II cells correlated with surfactant lipid and surfactant apoprotein expression. Am J Physiol 260: L562–L570. doi: 10.1152/ajplung.1991.260.6.L562 2058697

[23] Schellhase DE, Emrie PA, Fisher JH, Shannon JM (1989) Ontogeny of surfactant apoproteins in the rat. Pediatr Res 26: 167–174. doi: 10.1203/00006450-198909000-00001 2587115

[24] Wang J, Souza P, Kuliszewski M, Tanswell AK, Post M (1994) Expression of surfactant proteins in embryonic rat lung. Am J Respir Cell Mol Biol 10: 222–229. doi: 10.1165/ajrcmb.10.2.7509164 7509164

[25] Lin Y, Lechner AJ (1990) Ultrastructural analysis of regional type II cell development within fetal and neonatal lungs. Am J Physiol 259: L359–L364. doi: 10.1152/ajplung.1990.259.6.L359 2260670

[26] Hallman M (2013) The surfactant system protects both fetus and newborn. Neonatology 103: 320–326. 000349994 [pii]; doi: 10.1159/000349994 23736009

[27] Hawgood S, Poulain FR (2001) The pulmonary collectins and surfactant metabolism. Annu Rev Physiol 63: 495–519. doi: 10.1146/annurev.physiol.63.1.495 63/1/495 [pii]. 11181965

[28] Wright JR (2006) The "wisdom" of lung surfactant: balancing host defense and surface tension-reducing functions. Am J Physiol Lung Cell Mol Physiol 291: L847–L850. 00261.2006 [pii]; doi: 10.1152/ajplung.00261.2006 16861381

[29] Weaver TE, Conkright JJ (2001) Function of surfactant proteins B and C. Annu Rev Physiol 63: 555–578. doi: 10.1146/annurev.physiol.63.1.555 63/1/555 [pii]. 11181967

[30] Schmiedl A, Vieten G, Muhlfeld C, Bernhard W (2007) Distribution of intracellular and secreted surfactant during postnatal rat lung development. Pediatr Pulmonol 42: 548–562. doi: 10.1002/ppul.20623 17469149

[31] Smith PK, Krohn RI, Hermanson GT, Mallia AK, Gartner FH, Provenzano MD, et al. (1985) Measurement of protein using bicinchoninic acid. Anal Biochem 150: 76–85. 0003-2697(85)90442-7 [pii]; doi: 10.1016/0003-2697(85)90442-7 3843705

[32] Scherle W (1970) A simple method for volumetry of organs in quantitative stereology. Mikroskopie 26: 57–60. 5530651

[33] Schmiedl A, Hoymann HG, Ochs M, Menke A, Fehrenbach A, Krug N, et al. (2003) Increase of inactive intra-alveolar surfactant subtypes in lungs of asthmatic Brown Norway rats. Virchows Arch 442: 56–65. doi: 10.1007/s00428-002-0720-z 12536315

[34] Hsia CC, Hyde DM, Ochs M, Weibel ER (2010) An official research policy statement of the American Thoracic Society/European Respiratory Society: standards for quantitative assessment of lung structure. Am J Respir Crit Care Med 181: 394–418. 181/4/394 [pii]; doi: 10.1164/rccm.200809-1522ST 20130146 PMC5455840

[35] Schmiedl A, Wagener I, Jungen M, von HS, Stephan M (2021) Lung development and immune status under chronic LPS exposure in rat pups with and without CD26/DPP4 deficiency. Cell Tissue Res 386: 617–636. [pii];3522 [pii]; doi: 10.1007/s00441-021-03522-8 34606000 PMC8595150

[36] Schmiedl A, Luhrmann A, Pabst R, Koslowski R (2009) Increased surfactant protein a and d expression in acute ovalbumin-induced allergic airway inflammation in brown norway rats. Int Arch Allergy Immunol 148: 118–126. 000155742 [pii]; doi: 10.1159/000155742 18802356

[37] Schmiedl A, Roolfs T, Tutdibi E, Gortner L, Monz D (2017) Influence of prenatal hypoxia and postnatal hyperoxia on morphologic lung maturation in mice. PLOS ONE 12: e0175804. doi: 10.1371/journal.pone.0175804 PONE-D-16-26371 [pii]. 28426693 PMC5398543

[38] Appuhn SV, Siebert S, Myti D, Wrede C, Surate Solaligue DE, Perez-Bravo D, et al. (2021) Capillary Changes Precede Disordered Alveolarization in a Mouse Model of Bronchopulmonary Dysplasia. Am J Respir Cell Mol Biol 65: 81–91. doi: 10.1165/rcmb.2021-0004OC 33784484

[39] Massaro D, Teich N, Massaro GD (1986) Postnatal development of pulmonary alveoli: modulation in rats by thyroid hormones. Am J Physiol 250: R51–R55. doi: 10.1152/ajpregu.1986.250.1.R51 3942254

[40] Hyde DM, Blozis SA, Avdalovic MV, Putney LF, Dettorre R, Quesenberry NJ, et al. (2007) Alveoli increase in number but not size from birth to adulthood in rhesus monkeys. Am J Physiol Lung Cell Mol Physiol 293: L570–L579. 00467.2006 [pii]; doi: 10.1152/ajplung.00467.2006 17586691

[41] Pozarska A, Rodriguez-Castillo JA, Surate Solaligue DE, Ntokou A, Rath P, Mizikova I, et al. (2017) Stereological monitoring of mouse lung alveolarization from the early postnatal period to adulthood. Am J Physiol Lung Cell Mol Physiol 312: L882–L895. ajplung.00492.2016 [pii]; doi: 10.1152/ajplung.00492.2016 28314804

[42] Bersani I, Speer CP, Kunzmann S (2012) Surfactant proteins A and D in pulmonary diseases of preterm infants. Expert Rev Anti Infect Ther 10: 573–584. doi: 10.1586/eri.12.34 22702321

[43] Ledford JG, Pastva AM, Wright JR (2010) Review: Collectins link innate and adaptive immunity in allergic airway disease. Innate Immun 16: 183–190. 1753425910368446 [pii]; doi: 10.1177/1753425910368446 20418258 PMC3638252

[44] Ogasawara Y, Kuroki Y, Shiratori M, Shimizu H, Miyamura K, Akino T (1991) Ontogeny of surfactant apoprotein D, SP-D, in the rat lung. Biochim Biophys Acta 1083: 252–256. 0005-2760(91)90079-W [pii]; doi: 10.1016/0005-2760(91)90079-w 2049389

[45] Ohashi T, Pinkerton K, Ikegami M, Jobe AH (1994) Changes in alveolar surface area, surfactant protein A, and saturated phosphatidylcholine with postnatal rat lung growth. Pediatr Res 35: 685–689. doi: 10.1203/00006450-199406000-00013 7936819

[46] Wright JR (2005) Immunoregulatory functions of surfactant proteins. Nat Rev Immunol 5: 58–68. nri1528 [pii]; doi: 10.1038/nri1528 15630429

[47] Pastva AM, Wright JR, Williams KL (2007) Immunomodulatory roles of surfactant proteins A and D: implications in lung disease. Proc Am Thorac Soc 4: 252–257. 4/3/252 [pii]; doi: 10.1513/pats.200701-018AW 17607008 PMC2647627

[48] Wright JR (2004) Host defense functions of pulmonary surfactant. Biol Neonate 85: 326–332. doi: 10.1159/000078172 78172 [pii]. 15211087

[49] Mukherjee S, Giamberardino C, Thomas JM, Gowdy K, Pastva AM, Wright JR (2012) Surfactant protein A modulates induction of regulatory T cells via TGF-beta. J Immunol 188: 4376–4384. jimmunol.1101775 [pii]; doi: 10.4049/jimmunol.1101775 22474025 PMC3331948

[50] Herr C, Bals R (2007) [Innate immunity—species variation and development]. Pneumologie 61: 483–485. doi: 10.1055/s-2007-959224 17566960

[51] LeVine AM, Hartshorn K, Elliott J, Whitsett J, Korfhagen T (2002) Absence of SP-A modulates innate and adaptive defense responses to pulmonary influenza infection. Am J Physiol Lung Cell Mol Physiol 282: L563–L572. doi: 10.1152/ajplung.00280.2001 11839553

[52] Francisco D, Wang Y, Conway M, Hurbon AN, Dy ABC, Addison KJ, et al. (2020) Surfactant Protein-A Protects against IL-13-Induced Inflammation in Asthma. J Immunol 204: 2829–2839. jimmunol.1901227 [pii]; doi: 10.4049/jimmunol.1901227 32245819 PMC7304346

[53] Voorhout WF, Veenendaal T, Kuroki Y, Ogasawara Y, van Golde LM, Geuze HJ (1992) Immunocytochemical localization of surfactant protein D (SP-D) in type II cells, Clara cells, and alveolar macrophages of rat lung. J Histochem Cytochem 40: 1589–1597. doi: 10.1177/40.10.1527377 1527377

[54] Schmiedl A, Tschernig T, Brasch F, Pabst R, Bargsten G (2005) Decrease of the surface fraction of surfactant proteins containing clara cells and type II pneumocytes in a rat asthma model. Exp Toxicol Pathol 56: 265–272. S0940-2993(04)00066-1 [pii]; doi: 10.1016/j.etp.2004.10.004 15816355

[55] Auten RL, Watkins RH, Shapiro DL, Horowitz S (1990) Surfactant apoprotein A (SP-A) is synthesized in airway cells. Am J Respir Cell Mol Biol 3: 491–496. doi: 10.1165/ajrcmb/3.5.491 2223103

[56] Ochs M, Johnen G, Muller KM, Wahlers T, Hawgood S, Richter J, et al. (2002) Intracellular and intraalveolar localization of surfactant protein A (SP-A) in the parenchymal region of the human lung. Am J Respir Cell Mol Biol 26: 91–98. doi: 10.1165/ajrcmb.26.1.4570 11751208

[57] Fehrenbach H, Tews S, Fehrenbach A, Ochs M, Wittwer T, Wahlers T, et al. (2005) Improved lung preservation relates to an increase in tubular myelin-associated surfactant protein A. Respir Res 6: 60. 1465-9921-6-60 [pii]; doi: 10.1186/1465-9921-6-60 15969762 PMC1187923

[58] Sano H, Kuroki Y (2005) The lung collectins, SP-A and SP-D, modulate pulmonary innate immunity. Mol Immunol 42: 279–287. S0161-5890(04)00288-3 [pii]; doi: 10.1016/j.molimm.2004.07.014 15589315

[59] Hawgood S, Latham D, Borchelt J, Damm D, White T, Benson B, et al. (1993) Cell-specific posttranslational processing of the surfactant-associated protein SP-B. Am J Physiol 264: L290–L299. doi: 10.1152/ajplung.1993.264.3.L290 8460718

[60] Lin S, Na CL, Akinbi HT, Apsley KS, Whitsett JA, Weaver TE (1999) Surfactant protein B (SP-B) -/- mice are rescued by restoration of SP-B expression in alveolar type II cells but not Clara cells. J Biol Chem 274: 19168–19174. S0021-9258(19)74134-9 [pii]; doi: 10.1074/jbc.274.27.19168 10383422

[61] Seaborn T, Simard M, Provost PR, Piedboeuf B, Tremblay Y (2010) Sex hormone metabolism in lung development and maturation. Trends Endocrinol Metab 21: 729–738. S1043-2760(10)00143-8 [pii]; doi: 10.1016/j.tem.2010.09.001 20971653

[62] Beyer C, Kuppers E, Karolczak M, Trotter A (2003) Ontogenetic expression of estrogen and progesterone receptors in the mouse lung. Biol Neonate 84: 59–63. 71445 [pii]; doi: 10.1159/000071445 12890938

[63] Bresson E, Seaborn T, Cote M, Cormier G, Provost PR, Piedboeuf B, et al. (2010) Gene expression profile of androgen modulated genes in the murine fetal developing lung. Reprod Biol Endocrinol 8: 2. 1477-7827-8-2 [pii]; doi: 10.1186/1477-7827-8-2 20064212 PMC2822783

[64] Trotter A, Hilgendorff A, Kipp M, Beyer C, Kueppers E, Kiossis E, et al. (2009) Gender-related effects of prenatal administration of estrogen and progesterone receptor antagonists on VEGF and surfactant-proteins and on alveolarisation in the developing piglet lung. Early Hum Dev 85: 353–359. S0378-3782(08)00655-5 [pii]; doi: 10.1016/j.earlhumdev.2008.12.013 19186013

