# Correction: The expression of the surfactant proteins SP-A and SP-B during postnatal alveolarization of the rat lung

**DOI:** 10.1371/journal.pone.0329954

**Published:** 2025-08-06

**Authors:** Franziska Roeder, Lars Knudsen, Andreas Schmiedl

There is an error in affiliation 1 for all the authors. The correct affiliation 1 is: Institute of Functional and Applied Anatomy, Hannover Medical School, Hannover, Germany
