# Supplementary material for: The expression of the surfactant proteins SP-A and SP-B during postnatal alveolarization of the rat lung
Source: PLoS One. 2024 Mar 14;19(3):e0297889. doi: 10.1371/journal.pone.0297889 (PMC10939297; doi:10.1371/journal.pone.0297889)
Supplement: S1 File — (PDF) [file pone.0297889.s001.pdf]

Airspace density [%]

3 d	7 d	14 d	21 d	90 d
72,02	76,24	70,27	76,48	87,38
73,31	69,77	76,19	83,66	82,97
74,28	72,03	78,17	74,58	89,26
72,32	72,26	71,89	84,11	85,07
68,1	66,39	73,4	78,13	81,77
65,52	66,07	77,63	73,68	78,98

Septal volume density [%]

3d	7d	14d	21d	90d
27,98	23,76	29,73	23,91	12,62
26,68	30,23	23,81	16,34	17,03
25,72	27,97	21,83	25,42	10,74
27,68	27,74	28,11	15,89	14,93
31,9	33,62	26,6	21,88	18,23
34,48	33,93	22,37	26,32	21,02

Septal surface density 1/[µm]

3 d	7 d	14 d	21 d	90 d
0,03	0,0282	0,0318	0,0368	0,0441
0,028	0,028	0,0364	0,045	0,039
0,0289	0,0338	0,037	0,0396	0,0367
0,0315	0,031	0,0375	0,0456	0,0457
0,0328	0,031	0,0387	0,0369	0,041
0,029	0,0296	0,0386	0,0365	0,0364

Septal thickness [µm]

3 d	7 d	14 d	21 d	90 d
18,41	16,87	18,714	12,989	5,66
18,96	21,56	13,07	7,25	9,3
19,97	16,24	11,758	12,83	5,85
17,56	17,72	15,37	6,97	6,53
18,06	22,56	13,76	11,87	8,92
22,94	22,9	11,625	6,54	11,55

Airspace volume [cubic centimeters]

3 d	7 d	14 d	21 d	90 d
0,1629	0,2396	0,3414	0,5599	1,87105
0,1511	0,2509	0,3252	0,6552	2,34447
0,1285	0,2662	0,4536	0,5348	2,895
0,1676	0,2286	0,2753	0,6902	2,901
0,1274	0,3141	0,3294	0,6292	2,29
0,116	0,2217	0,3901	0,5792	1,2887

Septal volume [cubic centimeters]

3d	7d	14d	21d	90d
0,06329	0,07466	0,14445	0,17504	0,27023
0,05499	0,10869	0,10164	0,12531	0,48114
0,0445	0,10335	0,09903	0,18229	0,34835
0,06414	0,08778	0,10766	0,1304	0,50904
0,0597	0,15906	0,11938	0,17622	0,5101
0,06104	0,11387	0,11241	0,20691	0,34299

Septal surface [square centimeters]

3 d	7 d	14 d	21 d	90 d
67,86	88,61	154,51	269,51	944,31
57,71	100,67	155,381	352,44	1102,02
49,48	124,89	167,85	283,98	1190,36
72,99	98,1	143,63	374,2	1558,17
61,38	146,66	173,69	297,18	1147,23
51,34	99,34	193,97	286,94	593,94
