# Supplementary material for: The expression of the surfactant proteins SP-A and SP-B during postnatal alveolarization of the rat lung
Source: PLoS One. 2024 Mar 14;19(3):e0297889. doi: 10.1371/journal.pone.0297889 (PMC10939297; doi:10.1371/journal.pone.0297889)
Supplement: S2 File — (PDF) [file pone.0297889.s002.pdf]

relative surface of SP-A labeled AE II [%]

3 d	7 d	14 d	21 d	90 d
9,18	6	8,31	8,08	9,5
12,3	6,79	9,41	7,18	5,59
13,3	6,71	4,47	7,74	7,2
11,53	6,96	10,71	6,87	8,4
11,37	7,14	9,4	6,72	8,86
10,04	6,95	9,3	7,84	8,23

relative surfa ace of SP-B labeled AEII

3 d	7 d	14 d	21 d	90 d
4,35	3,77	4,6	3,79	4,26
4,05	5,41	4,88	3,48	4,49
3,11	4,08	3,46	3,98	5,15
4,57	4,46	5,27	4,73	7,05
2,92	4,16	4,03	4,51	5,99
2,83	3,61	4,36	5,05	4,49

SP-A gene expression

3 d	7 d	14 d	21 d	90 d
1,49	1,03	1,69	0,05	-1,02
1,47	1,29	1,85	-0,6	-1,43
1,54	1,49	1,51	-0,55	-1,29
1,54	0,92	1,36	0,02	-1,21
2,57	1,46	1,68	0	-0,92
2,43	1,79	1,6	0,05	-1,34
2,18	1,26		0,46	-1,5
			0,19	-1,39
			0,14	

SP-B gene expression

3 d	7 d	14 d	21 d	90 d
3,08	1,83	2,48	2,22	2,72
2,34	1,02	1,2	2,19	2,71
2,05	1,27	1,56	2,14	3,31
2,2	1,42	1,68	2	2,72
2,87	1,31	0,97	2,19	3,37
1,11	1,76	1,08	1,98	3,33
1,09		1,36	1,72	2,89
0,94			2,2	2,76
			2,73	

SP-A protein expression

3 d	7 d	14 d	21 d	90 d
448324	3276438	2379617	12292840	1233849
5022347	2135307	5923645	648587	659530
3076879	2256806	3450634	1157316	110463
467839,3	602883,4	1687155	39216	64838
2150449	939901,5	867373	33863	
692829,9	2910334	488294	24164	

SP-B Protein expression

3 d	7 d	14 d	21 d	90 d
2091945	6132980	3344050	13841100	9472368
4105355	6590435	6178674	11557820	9532572
5260010	4401661	8473275	10664570	8228873
1468275	8075694	8539841	7105610	5044620
7829723	6017791	7202807	8116349	
5670628	6579089	8176358	20457420	

SP-A gene expression related to adults

3 d	7 d	14 d	21 d
6,73	7,76	4,88	2,49
6,63	8,62	5,86	1,58
6,96	6,81	6,74	1,63
6,96	6,14	4,54	2,43
14,24	7,66	6,6	2,4
12,95	7,25	8,3	2,49
10,86		5,73	3,3
			2,73
			2,65

SP-B gene expression related to adults

3 d	7 d	14 d	21 d
0,11	-1,14	-0,5	-0,75
-0,63	-1,96	-1,78	-0,78
-0,92	-1,7	-1,42	-0,84
-0,77	-1,56	-1,3	-0,98
-0,1	-1,66	-2,01	-0,78
-1,86	-1,21	-1,9	-0,99
-1,88		-1,62	-1,25
-2,04		-2,64	-0,77
			-0,25
