# Supplementary material for: The expression of the surfactant proteins SP-A and SP-B during postnatal alveolarization of the rat lung
Source: PLoS One. 2024 Mar 14;19(3):e0297889. doi: 10.1371/journal.pone.0297889 (PMC10939297; doi:10.1371/journal.pone.0297889)
Supplement: S3 File — (PDF) [file pone.0297889.s003.pdf]

SV septa	P-A labeled AE II
0,03	9,18
0,028	12,3
0,0289	13,3
0,0315	11,53
0,0328	11,37
0,029	10,04
0,0282	6
0,028	6,79
0,0338	6,71
0,031	6,96
0,031	7,14
0,0296	6,95
0,0318	8,31
0,0364	9,41
0,037	4,47
0,0375	10,71
0,0387	9,4
0,0386	9,3
0,0368	8,08
0,045	7,18
0,0396	7,74
0,0456	6,87
0,0369	6,72
0,0365	7,84
0,0441	9,5
0,039	5,59
0,0367	7,2
0,0457	8,4
0,041	8,86
0,0364	8,23

S septa	P-A labeled AE II
67,86	9,18
57,71	12,3
49,48	13,3
72,99	11,53
61,38	11,37
51,34	10,04
88,61	6
100,67	6,79
124,89	6,71
98,1	6,96
146,66	7,14
99,34	6,95
154,51	8,31
155,381	9,41
167,85	4,47
143,63	10,71
173,69	9,4
193,97	9,3
269,51	8,08
352,44	7,18
283,98	7,74
374,2	6,87
297,18	6,72
286,94	7,84
944,31	9,5
1102,02	5,59
1190,36	7,2
1558,17	8,4
1147,23	8,86
593,94	8,23

SV septa	P-B labeled AE II	total surface sep	P-B labeled AE
0,03	4,35	67,86	4,35
0,028	4,05	57,71	4,05
0,0289	3,11	49,48	3,11
0,0315	4,57	72,99	4,57
0,0328	2,92	61,38	2,92
0,029	2,83	51,34	2,83
0,0282	3,77	88,61	3,77
0,028	5,41	100,67	5,41
0,0338	4,08	124,89	4,08
0,031	4,46	98,1	4,46
0,031	4,16	146,66	4,16
0,0296	3,61	99,34	3,61
0,0318	4,6	154,51	4,6
0,0364	4,88	155,381	4,88
0,037	3,46	167,85	3,46
0,0375	5,27	143,63	5,27
0,0387	4,03	173,69	4,03
0,0386	4,36	193,97	4,36
0,0368	3,79	269,51	3,79
0,045	3,48	352,44	3,48
0,0396	3,98	283,98	3,98
0,0456	4,73	374,2	4,73
0,0369	4,51	297,18	4,51
0,0365	5,05	286,94	5,05
0,0441	4,26	944,31	4,26
0,039	4,49	1102,02	4,49
0,0367	5,15	1190,36	5,15
0,0457	7,05	1558,17	7,05
0,041	5,99	1147,23	5,99
0,0364	4,49	593,94	4,49

II
