# Supplementary material for: The expression of the surfactant proteins SP-A and SP-B during postnatal alveolarization of the rat lung
Source: PLoS One. 2024 Mar 14;19(3):e0297889. doi: 10.1371/journal.pone.0297889 (PMC10939297; doi:10.1371/journal.pone.0297889)
Supplement: S4 File — (PDF) [file pone.0297889.s004.pdf]

SV septa	protein expression
0,03	448324
0,028	5022347
0,0289	3076879
0,0315	467839,3
0,0328	2150449
0,029	692829,9
0,0282	3276438
0,028	2135307
0,0338	2256806
0,031	602883,4
0,031	939901,5
0,0296	2910334
0,0318	2379617
0,0364	5923645
0,037	3450634
0,0375	1687155
0,0387	867373
0,0386	488294
0,0368	12292840
0,045	648587
0,0396	1157316
0,0456	39216
0,0369	33863
0,0365	24164
0,0441	1233849
0,039	659530
0,0367	110463
0,0457	64838

SV septa	protein expression
67,86	448324
57,71	5022347
49,48	3076879
72,99	467839,3
61,38	2150449
51,34	692829,9
88,61	3276438
100,67	2135307
124,89	2256806
98,1	602883,4
146,66	939901,5
99,34	2910334
154,51	2379617
155,381	5923645
167,85	3450634
143,63	1687155
173,69	867373
193,97	488294
269,51	12292840
352,44	648587
283,98	1157316
374,2	39216
297,18	33863
286,94	24164
944,31	1233849
1102,02	659530
1190,36	110463
1558,17	64838

SV septa	protein expression
0,03	2091945
0,028	4105355
0,0289	5260010
0,0315	1468275
0,0328	7829723
0,029	5670628
0,0282	6132980
0,028	6590435
0,0338	4401661
0,031	8075694
0,031	6017791
0,0296	6579089
0,0318	3344050
0,0364	6178674
0,037	8473275
0,0375	8539841
0,0387	7202807
0,0386	8176358
0,0368	13841100
0,045	11557820
0,0396	10664570
0,0456	7105610
0,0369	8116349
0,0365	20457420
0,0441	9472368
0,039	9532572
0,0367	8228873
0,0457	5044620

SV septa	protein expres
67,86	2091945
57,71	4105355
49,48	5260010
72,99	1468275
61,38	7829723
51,34	5670628
88,61	6132980
100,67	6590435
124,89	4401661
98,1	8075694
146,66	6017791
99,34	6579089
154,51	3344050
155,381	6178674
167,85	8473275
143,63	8539841
173,69	7202807
193,97	8176358
269,51	13841100
352,44	11557820
283,98	10664570
374,2	7105610
297,18	8116349
286,94	20457420
944,31	9472368
1102,02	9532572
1190,36	8228873
1558,17	5044620

ssion
