# Supplementary material for: The expression of the surfactant proteins SP-A and SP-B during postnatal alveolarization of the rat lung
Source: PLoS One. 2024 Mar 14;19(3):e0297889. doi: 10.1371/journal.pone.0297889 (PMC10939297; doi:10.1371/journal.pone.0297889)
Supplement: S5 File — (PDF) [file pone.0297889.s005.pdf]

SV septa	mRNA Expression
0,03	1,49
0,028	1,47
0,0289	1,54
0,0315	1,54
0,0328	2,57
0,029	2,43
0,0282	1,03
0,028	1,29
0,0338	1,49
0,031	0,92
0,031	1,46
0,0296	1,79
0,0318	1,69
0,0364	1,85
0,037	1,51
0,0375	1,36
0,0387	1,68
0,0386	1,6
0,0368	0,05
0,045	-0,6
0,0396	-0,55
0,0456	0,02
0,0369	0
0,0365	0,05
0,0441	-1,02
0,039	-1,43
0,0367	-1,29
0,0457	-1,21
0,041	-0,92
0,0364	-1,34

total surface sep	mRNA Expression
67,86	1,49
57,71	1,47
49,48	1,54
72,99	1,54
61,38	2,57
51,34	2,43
88,61	1,03
100,67	1,29
124,89	1,49
98,1	0,92
146,66	1,46
99,34	1,79
154,51	1,69
155,381	1,85
167,85	1,51
143,63	1,36
173,69	1,68
193,97	1,6
269,51	0,05
352,44	-0,6
283,98	-0,55
374,2	0,02
297,18	0
286,94	0,05
944,31	-1,02
1102,02	-1,43
1190,36	-1,29
1558,17	-1,21
1147,23	-0,92
593,94	-1,34

SV septa	RNA Expression	SV septa	RNA Expressio
0,03	3,08	67,86	3,08
0,028	2,34	57,71	2,34
0,0289	2,05	49,48	2,05
0,0315	2,2	72,99	2,2
0,0328	2,87	61,38	2,87
0,029	1,11	51,34	1,11
0,0282	1,83	88,61	1,83
0,028	1,02	100,67	1,02
0,0338	1,27	124,89	1,27
0,031	1,42	98,1	1,42
0,031	1,31	146,66	1,31
0,0296	1,76	99,34	1,76
0,0318	2,48	154,51	2,48
0,0364	1,2	155,381	1,2
0,037	1,56	167,85	1,56
0,0375	1,68	143,63	1,68
0,0387	1,36	173,69	0,97
0,0386	1,08	193,97	1,08
0,0368	2,22	269,51	2,22
0,045	2,19	352,44	2,19
0,0396	2,14	283,98	2,14
0,0456	2	374,2	2
0,0369	2,19	297,18	2,19
0,0365	1,98	286,94	1,98
0,0441	2,72	944,31	2,72
0,039	2,71	1102,02	2,71
0,0367	3,31	1190,36	3,31
0,0457	2,72	1558,17	2,72
0,041	3,37	1147,23	3,37
0,0364	3,33	593,94	3,33

on
